# Causal inference between serum bilirubin levels and juvenile idiopathic arthritis‐associated uveitis: A bidirectional Mendelian randomization study

**DOI:** 10.1002/hsr2.1847

**Published:** 2024-02-01

**Authors:** Jun Zhang, Peng Zhou, Shuqiong Hu, Shiya Cai, Tao He

**Affiliations:** ^1^ Department of Ophthalmology Renmin Hospital of Wuhan University Wuhan Hubei China; ^2^ College of Architecture Changsha University of Science and Technology Changsha Hunan China; ^3^ Wuhan Aier Eye Hospital of Wuhan University Wuhan Hubei China; ^4^ Department of Ophthalmology, The First Affiliated Hospital Zhejiang University School of Medicine Hangzhou Zhejiang China

**Keywords:** bilirubin, causal inference, JIA associated uveitis, Mendelian randomization, single‐nucleotide polymorphism

## Abstract

**Background:**

Several observational studies have suggested an association between low serum bilirubin levels and Behçet's disease uveitis. However, the causal inference between bilirubin level and juvenile idiopathic arthritis‐associated uveitis (JIAU) remains ambiguous. We investigated the potential causal relationship between serum bilirubin levels and JIAU using a bidirectional two‐sample Mendelian randomization (MR) framework.

**Methods:**

We systemically integrated summary‐level data from published large‐scale genome‐wide association studies on bilirubin level and JIAU in a Caucasian British population. To determine the causal effect of bilirubin level on JIAU, we constructed strong instrumental variables using 47 and 80 single‐nucleotide polymorphisms (SNPs) specific to direct bilirubin and total bilirubin levels, respectively. For reverse causal inference, seven SNPs associated with JIAU were included in our study. Multiple complementary methods were further performed to evaluate the robustness of MR estimates.

**Results:**

The inverse‐variance weighted estimate did not show any significant causal associations of genetically predicted serum direct or total bilirubin level with the risk of JIAU (odds ratio [OR]: 1.010, 95% confidence interval [CI]: 0.750–1.359, *p* = 0.947; OR: 0.867, 95% CI: 0.688–1.093; *p* = 0.227, respectively). MR–Egger and weighted median methods also obtained similar associations. Additionally, the results of reverse MR analyses using JIAU as exposure showed no associations of genetically predicted risk of JIAU with serum bilirubin levels (*p* > 0.05). In sensitivity analysis, the causal estimate between serum bilirubin levels and JIAU did not differ when SNPs associated with possible confounders were omitted.

**Conclusion:**

Genetic evidence from our bidirectional analysis did not support a causal association between serum bilirubin levels and JIAU risk in the Caucasian British population. Future large‐scale studies should be conducted to validate these findings and explore any causal effects on the disease process.

## 1 INTRODUCTION

Although bilirubin has traditionally been considered a secondary excretory product of heme metabolism and is harmful to human physiology,^1^ it has recently been recognized as an immunomodulatory molecule with potent antioxidant and anti‐inflammatory activities.^2^, ^3^ Its plasma concentration in humans usually ranges from 5 to 17 µmol/L under physiological conditions.^4^ Experimental and human studies have reported a correlation between relatively high total serum bilirubin levels and a reduced risk of CD4^+^ T cell‐related autoimmune disorders, such as multiple sclerosis, systemic lupus erythematosus, rheumatoid arthritis, and inflammatory bowel disease.^5^, ^6^, ^7^, ^8^ It has also been pointed out that decreased serum bilirubin levels have also been shown to contribute to aggravated inflammation in patients with Behçet disease‐related uveitis.^9^ Juvenile idiopathic arthritis‐associated uveitis (JIAU), a form of anterior uveitis, is mediated by immune‐related pathways and mechanisms.^10^, ^11^ To our knowledge, no cohort study in the literature has shown a true causal relationship between serum bilirubin levels and JIAU. Observational studies are potentially highly prone to bias owing to their small sample size and unobserved confounding factors, which would preclude a clear understanding of the causal relationship. Therefore, its exact effects remain unclear.

The confusing correlation between the two clinical phenotypes due to several shared factors presents a challenge for the confirmation of their causal inference. Mendelian randomization (MR) is a powerful analytical approach for estimating the causal effects of exposure on an outcome.^12^ Compared with conventional observational research, MR analysis is less susceptible to reverse causality, potential confounding bias, and measurement error.^13^ Two‐sample MR analysis is an extension of the MR method that only requires publicly available GWAS (genome‐wide association study) summary statistics, without directly referring to individual‐level data. Zhong et al. discovered the causal effects of tuberculosis exposure and serum 25‐hydroxyvitamin D levels on the risk of Behçet's uveitis.^14^, ^15^

To obtain robust and consistent conclusions, we applied recently published large‐scale GWASs of serum bilirubin (including direct and total bilirubin levels) and JIAU to evaluate the potential causal relationship between these two traits using a bidirectional two‐sample MR analysis. Thereafter, we utilized multiple MR methods and prioritized methods that are powerful for the validation of horizontal pleiotropy and the influence of outlying genetic instruments.

## 2 MATERIALS AND METHODS

### 2.1 Participants and data sources

We used the most recent GWASs with a phenotype specific to serum direct and total bilirubin levels, including 317,639 individuals of the Caucasian British population (European ancestry) from the UK Biobank (UKB).^16^ The UKB project has so far recruited approximately 500,000 people aged 40–69 years from 2006 to 2010 across the UK population.^17^ Confounding factors, such as age, sex, the top 40 principal components for population stratification, indicators of socioeconomic status, recruitment center, and potential technical confounders (fasting time, blood sampling time, and sample dilution factor) were adjusted for the measurement of raw bilirubin levels.^16^ The association analysis was conducted for ~770,000 single‐nucleotide polymorphisms (SNPs) with a linear additive regression model. All summary statistics (effect allele, effect allele frequency, effect size, standard error, *p* value, and sample size) were downloaded and reorganized.

A GWAS summary data set for JIAU was acquired from the MRC IEU Open GWAS Project (https://gwas.mrcieu.ac.uk/). After stringent quality control, 94,197 European individuals (1430 cases and 92,767 controls) and more than 16,150,000 genotyped and imputed SNPs were reserved for JIAU. Additive logistic/linear regression was introduced to evaluate the association between each SNP and JIAU while adjusting for other available covariates (e.g., age and sex). Summary association statistics of JIAU (e.g., effect allele, effect allele frequency, effect size, standard error, *p* value, and sample size) are also available. The GWAS genetic data sets used in this study are summarized in Table 1.

**Table 1:** Genetic data sets used in the current study.

Exposure (outcome)	Datasource	Sample size	Number of SNPs	PMID
Direct bilirubin	UK Biobank	317,639	779,718	33462484
Total bilirubin	UK Biobank	317,639	780,395	33462484
JIAU	FinnGen Biobank	94,197	16,152,119	NA

Abbreviations: JIAU, Juvenile idiopathic arthritis‐associated uveitis; NA, not available; SNP, single‐nucleotide polymorphism.

The selected participants in these two large‐scale GWAS studies were mostly of European ancestry. Written informed consent was obtained from all corresponding original studies. Each study was approved by the local institutional review board and ethics committee.

### 2.2 MR assumptions

**Figure 1:**
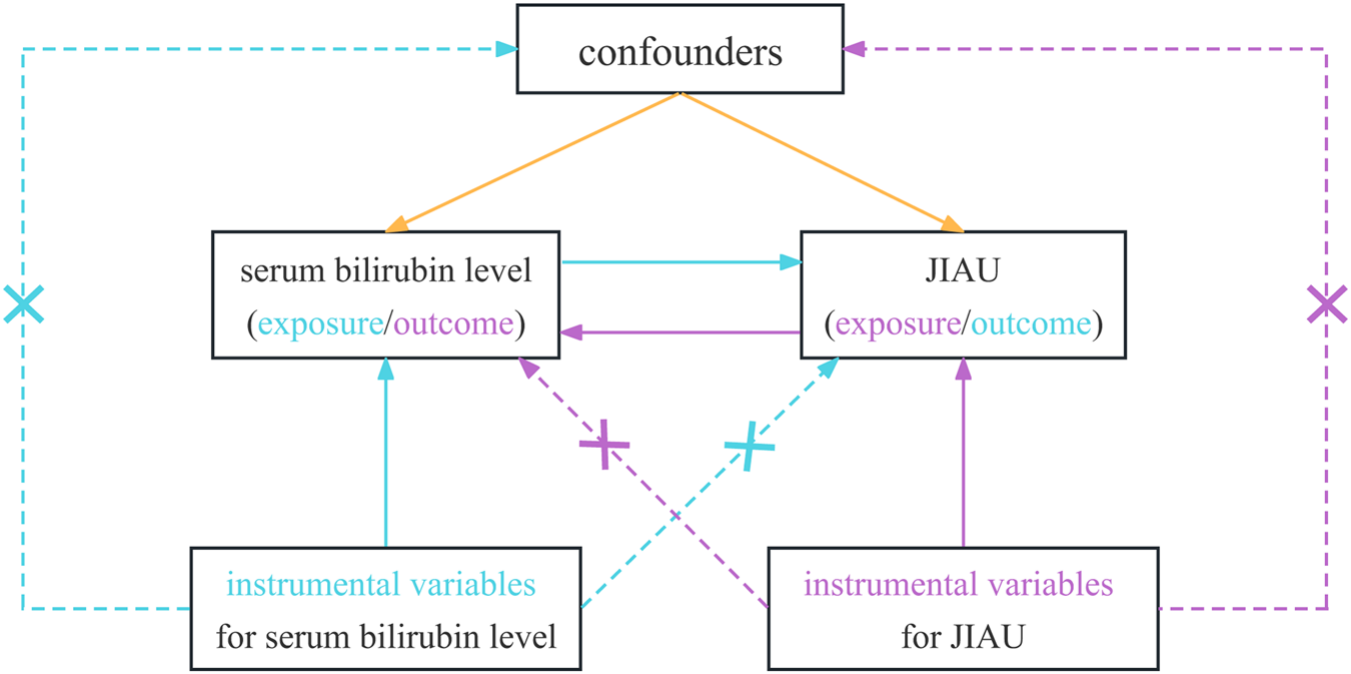
Schematic representation of the bidirectional MR analyses with required instrumental variables. The arrows show the MR assumptions, such that the instrumental variables (SNPs) are correlated with the exposure factor, are not associated with confounding factors, and only affect the outcome through exposure. MR, Mendelian randomization; SNP, single‐nucleotide polymorphism.

We employed two nonoverlapping two‐sample MR analyses to test the potential causal inference between serum (direct and total) bilirubin levels and risk of JIAU in a bidirectional manner. To obtain reliable results, the valid genetic IVs in the MR analysis process must satisfy three pivotal assumptions (18): (a) the SNPs must be solidly correlated with the exposure (IV1); (b) the SNPs must be independent of any confounders affecting both exposure and outcome (IV2); and (c) the SNPs and the outcome must be associated only through the exposure (IV3). There would be a violation of assumptions IV2 and/or IV3 in MR analyses in the presence of horizontal pleiotropy (Figure 1).

### 2.3 Selection of genetic instrumental variables

All genetic IVs for the MR analysis were selected to fit the three MR assumptions described above. Genetic instruments for estimating the causal effect of bilirubin level on the risk of JIAU were obtained from a bilirubin‐related GWAS data set. A total of 996 index SNPs associated with direct bilirubin levels and 2710 with total bilirubin levels were associated with exposure at a genome‐wide significance level (*p* < 5.0 × 10^−8^). To investigate the causal effect of JIAU on bilirubin level, 4083 index SNPs that reported a significant relationship with JIAU (*p* < 5.0 × 10^−8^) were included as candidate genetic instruments.

To ensure the independence of genetic instruments, we applied a linkage disequilibrium clumping algorithm with clumping cutoffs setting 0.01 for *R* ^2^ and 5000 kb for physical distance as reference panel. The effect allele of each SNP in the summary statistics of both the exposure and outcome was screened using the data harmonization function, which could remove palindromic SNPs with ambiguous strand identification or opposite strand SNPs. We investigated the assumption that genetic IVs are independent of outcome and confounding factors (IV2 and IV3) by searching an online GWAS catalog at http://www.phenoscanner.medschl.cam.ac.u.k/.

### 2.4 Statistical analysis

Common MR approaches, including inverse variance weighted^18^ maximum likelihood,^19^ and weighted median regression^20^ have been employed to obtain MR effect estimates. To test the bias of horizontal pleiotropy, sensitivity analyses, such as MR–Egger and MR pleiotropy residual sum and outlier tests, were performed.^21^, ^22^ We conducted a multivariable MR analysis to adjust for potential confounding factors, including soluble intercellular adhesion molecule 1, C‐reactive protein levels, and autoimmune diseases. All analyses were repeated after the removal of palindromic SNPs to avoid ambiguous results. Since the genetic instruments are required to be solidly associated with exposure (IV1), we calculated the proportion of each phenotypic IV explained by the sample size and number of instruments using the *R* ^2^ statistic.^23^ Typically, index SNPs with a quantified *F* statistic >10 were defined as strong instruments and recommended for MR analyses.^24^ For reference, an *F* statistic of 10 in a two‐sample MR setting revealed a relative bias of 10% toward the null.^25^

Heterogeneity between genetic variants was assessed using Cochran's *Q* test, and a *p* value below 0.1 was interpreted as the presence of significant heterogeneity. The results for the outcomes or per predicted decrease/increase were expressed as an estimate or odds ratio (OR) with a 95% confidence interval (CI). All statistical tests were two‐sided, and *p* value < 0.05, indicating strong evidence of association in this study. All MR analyses were performed using *TwoSampleMR* and *MR‐PRESSO* packages in R environment version 4.1.0 (R Project for Statistical Computing).

## 3 RESULTS

### 3.1 Instrumental variables associated with serum bilirubin and JIA‐associated uveitis

Our statistical association tests for bilirubin and JIAU significantly predicted their separate phenotypes, suggesting their suitability for use as indicators of these traits. The exposure‐specific index SNPs were used as the IVs. Forty‐seven SNPs for direct bilirubin levels, 80 for total bilirubin levels, and 25 for JIAU were eligible for subsequent analysis. As calculated from the summary statistics, these SNPs could explain approximately 3.65% of the variance in direct bilirubin (*R* ^2^ = 0.0365), 7.59% in total bilirubin (*R* ^2^ = 0.0759), and 2.45% in JIAU (*R* ^2^ = 0.0245). All these independent SNPs constituted single instruments with F statistic ranging from 28.94 to 285.09, and with an overall F statistic of 97.82, suggesting that the selected SNPs have sufficiently strong power to be treated as IVs. In addition, the effect size and standard error of each SNP were obtained, and are summarized in detail in Supporting Information: Tables 1 and 2.

### 3.2 Causal association between serum bilirubin and JIA‐associated uveitis via MR analysis

Table 2 reports the MR effect estimates for serum bilirubin and JIAU using inverse variance‐weighted, maximum likelihood, and weighted median regression models. Random‐effects models were used because of the presence of substantial heterogeneity (*p* < 0.1). Using the conventional inverse variance weighted method, we did not find any evidence of a causal relationship between bilirubin levels and the risk of JIAU. Serum direct and total bilirubin levels were not associated with JIAU risk (direct bilirubin level, OR: 1.010, 95% CI: 0.750–1.359, *p* = 0.947; total bilirubin level, OR: 0.867, 95% CI: 0.688–1.093, *p* = 0.227). Consistently, the maximum likelihood and weighted median regressions indicated similar estimated associations that lacked adequate power to reach significance thresholds (direct bilirubin level, maximum likelihood, OR: 1.010, 95% CI: 0.814–1.253, *p* = 0.927; total bilirubin level, maximum likelihood, OR: 0.866, 95% CI: 0.710–1.058, *p* = 0.159). In addition, the removal of palindromic SNPs and adjustment for confounding traits, such as soluble intercellular adhesion molecule 1, C‐reactive protein level, and autoimmune diseases, also did not influence the findings (Table 2).

**Table 2:** Risk of bilirubin levels or JIAU in genetically susceptible individuals.

	Full‐set						Remove palindromic SNPs					
	*N*	OR	95% CI	*p* Value	*p* Value for hetero	*p* Value for inter	*N*	OR	95% CI	*p* Value	*p* Value for hetero	*p* Value for inter
Risk of bilirubin level in persons genetically susceptible to JIAU												
Direct bilirubin (exposure)												
IVW	47	1.01	0.750–1.359	0.947	<0.001		43	1.035	0.789–1.361	0.799	0.009	
Maximum likelihood	47	1.01	0.814–1.253	0.927	<0.001		43	1.036	0.834–1.287	0.749	0.009	
MR–Egger	47	1.254	0.853–1.842	0.255	<0.001	0.098	43	1.228	0.859–1.756	0.267	0.013	0.163
Weighted median	47	1.203	0.899–1.609	0.213			43	1.204	0.894–1.620	0.221		
Adjusted for ADs	45	1.033	0.791–1.349	0.812	0.013							
Adjusted for CRP	45	1.027	0.759–1.389	0.86	<0.001							
Adjusted for ICAM	46	1.01	0.748–1.365	0.947	<0.001							
Total bilirubin (exposure)												
IVW	80	0.867	0.688–1.093	0.227	0.02		75	0.841	0.654–1.082	0.178	0.009	
Maximum likelihood	80	0.866	0.710–1.058	0.159	0.02		75	0.84	0.679–1.039	0.107	0.009	
MR–Egger	80	0.855	0.640–1.143	0.294	0.017	0.877	75	0.826	0.603–1.130	0.236	0.008	0.847
Weighted median	80	1.062	0.778–1.450	0.705			75			0.614		
Adjusted for ADs	76	0.883	0.714–1.093	0.253	0.203							
Adjusted for CRP	77	0.892	0.711–1.119	0.325	0.051							
Adjusted for ICAM	79	0.867	0.687–1.095	0.23	0.017							
Risk of JIAU in persons genetically susceptible to bilirubin level												
Direct bilirubin (outcome)												
IVW	7	0.999	0.989–1.008	0.771	<0.001		6	1.004	0.989–1.019	0.56	0.001	
Maximum likelihood	7	0.998	0.994–1.003	0.557	<0.001		6	1.004	0.997–1.012	0.236	0.001	
MR–Egger	7	0.997	0.982–1.012	0.717	<0.001	0.794	6	1.008	0.980–1.037	0.597	<0.001	0.755
Weighted median	7	0.999	0.993–1.005	0.801			6	1.008	0.999–1.017	0.05		
Total bilirubin (outcome)												
IVW	7	0.997	0.988–1.005	0.407	0.001		6	1.004	0.993–1.015	0.483	0.015	
Maximum likelihood	7	0.996	0.992–1.001	0.109	0.001		6	1.004	0.997–1.011	0.235	0.016	
MR–Egger	7	0.994	0.981–1.007	0.382	<0.001	0.583	6	1.007	0.985–1.030	0.558	0.008	0.751
Weighted median	7	0.996	0.990–1.002	0.224			6	1.008	0.999–1.016	0.052		

Abbreviations: 95% CI, 95% confidence interval; AD, autoimmune disease; CRP, C‐reactive protein; ICAM, intercellular adhesion molecule; IVW, inverse variance weighted; JIAU, Juvenile idiopathic arthritis‐associated uveitis; N, number of SNPs; OR, odd ratio; p Value for hetero; p Value for heterogeneity; *p* Value for inter, *p* Value for intercept; SNP, single‐nucleotide polymorphism.

No significant association between JIAU and serum bilirubin levels was observed in the reverse direction with the implementation of inverse variance weighted, maximum likelihood, and weighted median regression models (Table 2, *p* > 0.05). MR‐related funnel plots were used to display the individual Wald ratios for a single SNP plotted against the precision. Horizontal pleiotropy was detected by the asymmetry in the funnel plot. Our assessment of funnel plots showed a symmetrical direction in the current MR analysis. In addition, the four MR–Egger intercepts did not reveal any evidence of significant directional pleiotropy (*p* = 0.098, *p* = 0.877, *p* = 0.794, and *p* = 0.583, respectively; Table 2). These results demonstrated that directional pleiotropic effects were not observed between serum bilirubin levels and JIAU in both directions.

### 3.3 Effects of individual genetic instruments between serum bilirubin and JIA‐associated uveitis

To further compare the effect of individual IV on the overall causal association, we performed leave‐one‐out analyses in our bidirectional MR analysis. In terms of the sensitivity analysis, we systematically removed each SNP and repeated the MR analyses, and there was no substantial difference in the estimated causal effect. It is likely that the estimated effects cannot be explained using a single genetic instrument. Both raw and outlier‐corrected estimates (excluding two SNPs for direct bilirubin, one SNP for total bilirubin, and three SNPs for JIAU) were in agreement with the leave‐one‐out and MR–Egger intercept analyses, which is not necessarily indicative of pleiotropic effects that could bias the overall analyses (*p* distortion >0.05; Table 3).

**Table 3:** MR‐PRESSO estimates between serum bilirubin levels and JIAU susceptibility.

	Raw estimates				Outlier corrected estimates				
Exposure versus outcome	*N*	OR	95% CI	*p* Value	*N*	OR	95% CI	*p* Value	*p* distortion
Direct bilirubin versus JIAU	47	1.01	0.750–1.359	0.948	45	1.067	0.871–1.308	0.535	0.797
Total bilirubin versus JIAU	80	0.867	0.688–1.093	0.23	79	0.889	0.720–1.097	0.276	0.771
JIAU versus direct bilirubin	7	0.998	0.989–1.008	0.781	4	1.002	0.980–1.024	0.875	0.280
JIAU versus total bilirubin	7	0.996	0.988–1.005	0.439	4	0.996	0.979–1.013	0.681	0.493

Abbreviations: 95% CI, 95% confidence interval; IVW, inverse variance weighted; JIAU, Juvenile idiopathic arthritis‐associated uveitis; N, number of SNPs; OR, odd ratio; SNP, single‐nucleotide polymorphism.

## 4 DISCUSSION

In this study, we conducted an MR analysis with multiple complementary MR methods to systemically investigate the role of serum bilirubin levels in JIAU and their reverse causal relationship using data from the most recent and largest GWAS summary statistics. Independent genetic variants (SNPs) with genome‐wide significance for bilirubin and JIAU were used as IVs, and all IVs constituted strong instruments with an *F* statistic >10. However, our study did not find strong evidence to support a causal genetic association between serum bilirubin levels and risk of JIAU in either direction.

In contrast to random control trials, MR studies are more feasible and robust to use genetic variants to assess the causality of nongenetic exposure or a modifiable risk factor of interest. Compared with their relatively short follow‐up time, MR may be better able to detect exposures that occur over a long period.^26^ The largest sample size of this kind, including more than 400,000 individuals of European ancestry (317,639 individuals for bilirubin and 94,197 for JIAU) in our current study greatly improved the accuracy and precision of MR estimates. Additionally, we performed the first bidirectional MR analysis of the causal relationship between serum bilirubin levels and autoimmune JIAU. Our two‐sample MR study was sufficiently powered to assess the causal associations between them.

Evidence for the association between serum bilirubin levels and multiple immune‐ or inflammation‐related disorders, including cardiovascular disease, stroke, nonalcoholic fatty liver disease, type 2 diabetes, and cancers, has been uncovered in several updated MR analyses.^27^, ^28^, ^29^, ^30^, ^31^ Observational research has confirmed that a low level of serum bilirubin is associated with an increased risk of autoimmune diseases such as Crohn's disease, ulcerative colitis, rheumatoid arthritis, systemic lupus erythematosus, and polymyositis.^32^, ^33^, ^34^, ^35^, ^36^ In a mouse model of experimental autoimmune encephalomyelitis, bilirubin mediated its immune response to CD4^+^ T cell reactivity via inhibition of its costimulatory activity and immune transcription factor activation.^37^, ^38^ These findings suggested that bilirubin could be used as a new therapeutic target for various CD4^+^ T cell‐associated autoimmune diseases.

It has been well documented that those autoimmune diseases often have a common immunological basis and molecular mechanism.^39^ In consistent with the aforementioned observational studies, serum bilirubin levels were estimated to be negatively correlated with Behçet's uveitis in the Turkish population.^9^ Despite the presentation of the function of bilirubin in various inflammation‐related diseases, no MR study has investigated bilirubin levels in patients with autoimmune JIAU. Therefore, we performed the present MR investigation to evaluate the relationship between bilirubin level and JIAU. Our study focused on the Caucasian British population instead of the previous observational research on Turkish ancestry. The reasons for these controversial results may be multifactorial. In our research, we only considered the presence or absence of JIAU and did not consider the stratification of its severity or duration, which may have contributed to our lack of positive findings. Observational studies have the potential to be plagued by a measurement error bias, relatively small sample sizes, various ethnic origins, confounding traits, and various disease states. Instead, MR studies utilize uninfluenced genetic polymorphisms as proxies for modifiable risk factors and outcomes independent of these biases. Moreover, MR studies exploring causality could decrease the costs of patient recruitment and save time in follow‐up work.

Several mechanisms may be involved in the association between bilirubin levels and autoimmune disease. Bilirubin can function as an inhibitor of the complement cascade by interrupting the binding of the complement C1 complex to antibodies, thus directly affecting innate immunity.^40^ Bilirubin can inhibit cell‐surface expression of the major histocompatibility complex II class molecule B7 on antigen‐presenting cells, thereby impairing antigen presentation to lymphocytes.^38^ Bilirubin is a potent endogenous activator of the aryl hydrocarbon receptor in the adaptive immune process and plays a pivotal role in the immune response, including modulation of the forkhead box protein 3 transcription factor expressed by regulatory T cells and differentiation of T‐helper 17 and B cells.^3^ In our MR study, multiple sensitivity analysis and MR pleiotropy residual sum and outlier test highlighted a robust conclusion between serum bilirubin and JIAU in two reversed directions. Despite insufficient evidence to support this association, it does not suggest that the supplementation of bilirubin‐like drugs in autoimmune JIAU is dispensable. Synthetic bilirubin nanoparticles centered on antioxidant and anti‐inflammatory properties could be used as therapeutic nanomedicines for multiple intravenous injections, as reported by Kim et al.^37^

The current MR analysis method offers several advantages. First, this study incorporated the largest and newest GWAS datasets of serum bilirubin levels and JIAU to obtain the genetic data. Only individuals of European ancestry were recruited to minimize the bias in population stratification.^41^ Second, we performed several rigorous sensitivity analyses to corroborate MR assumptions. The most significant independent SNPs were selected to guarantee their strong association with exposure of interest in two opposite directions. Third, IVs associated with horizontal confounding on the exposure‐outcome effect were further removed to satisfy the “exclusion restriction” assumption.^42^ Despite the use of updated GWAS datasets in our study, some potential limitations need to be noted. First, the average risk estimate of genetic variants for a specific trait considered the entire lifetime of the participants instead of a certain period of life. Thus, it cannot explain the effect on the risk of an outcome in any period. Second, the discovered exposure‐associated SNPs explained only 3.65%, 7.59%, and 2.45% of the phenotypic variance, which limits the evaluation of a tiny causality between the two traits. Third, confinement to the Caucasian British population of European ancestry in our MR analysis limits the universality of our findings. Fourth, the duration and severity of JIAU, the correlation with age of onset, and predictive parameters were not taken into account in this study, which also requires further investigation. In addition, general information on the basic medications affecting JIAU was not available. Further well‐conducted GWASs with larger sample sizes are required to further examine the potential etiological role of serum bilirubin levels in various diseases.

In summary, our large‐scale bidirectional two‐sample MR analysis does not provide sufficient evidence to support a causal inference between serum bilirubin levels and the risk of JIAU in both directions. Randomized clinical trials will be required to confirm whether serum bilirubin levels ultimately play a causal role in the etiology of JIAU and to determine if potential inhibition represents a novel therapeutic strategy for patients with JIAU.

## AUTHOR CONTRIBUTIONS

**Jun Zhang**: Conceptualization; data curation; formal analysis; funding acquisition. **Peng Zhou**: Methodology; software. **Shuqiong Hu**: Data curation; formal analysis; validation. **Shiya Cai**: Data curation; investigation; methodology. **Tao He**: Conceptualization; supervision; writing—review and editing.

## CONFLICT OF INTEREST STATEMENT

The authors declare no conflict of interest.

## ETHICS STATEMENT

All analyses were based on previously published studies; therefore, no ethical approval or patient consent was required.

## TRANSPARENCY STATEMENT

The lead author Tao He affirms that this manuscript is an honest, accurate, and transparent account of the study being reported; that no important aspects of the study have been omitted; and that any discrepancies from the study as planned (and, if relevant, registered) have been explained.

## Supporting information

Supporting information.

Supporting information.

## Data Availability

The data used in our Mendelian randomization analysis were obtained from the UK Biobank and the MRC IEU Open GWAS Project. The authors also acknowledge the participants and investigators of the FinnGen Study. Further inquiries can be directed to the corresponding author.
