# Supplementary material for: Causal inference between serum bilirubin levels and juvenile idiopathic arthritis‐associated uveitis: A bidirectional Mendelian randomization study
Source: Health Sci Rep. 2024 Feb 1;7(2):e1847. doi: 10.1002/hsr2.1847 (PMC10835017; doi:10.1002/hsr2.1847)
Supplement: Supplementary file 1 — Supporting information. [file HSR2-7-e1847-s002.docx]

Supplement Table 1. Summary statistics for MR analysis of the potential causal effect of serum bilirubin levels on JIA associated iridocyclitis.

| No. | SNP | Chr. | EA | OA | EAF | Exposure: direct bilirubin | | Exposure: total bilirubin | | Outcome: iridocyclitis | | R^2^ | F |
| --- | --- | --- | --- | --- | --- | --- | --- | --- | --- | --- | --- | --- | --- |
|  |  |  |  |  |  | Beta | SE | Beta | SE | Beta | SE |  |  |
| 1 | rs11045879 | 12 | C | T | 0.1899 | 0.1546 | 0.0034 |  |  | 0.0248 | 0.0414 | 1.26E-03 | 206.574 |
| 2 | rs111712352 | 2 | T | C | 0.0268 | -0.2665 | 0.0067 |  |  | 0.0068 | 0.1995 | 2.76E-04 | 158.140 |
| 3 | rs11187157 | 10 | C | T | 0.4274 | 0.0167 | 0.0026 |  |  | 0.0138 | 0.0389 | 1.25E-03 | 41.256 |
| 4 | rs113732002 | 2 | C | A | 0.0636 | -0.0272 | 0.0048 |  |  | 0.0388 | 0.0778 | 6.21E-04 | 32.111 |
| 5 | rs114252547 | 2 | G | T | 0.0328 | 0.2539 | 0.0069 |  |  | 0.0416 | 0.1217 | 7.58E-04 | 135.027 |
| 6 | rs114667609 | 2 | G | T | 0.0189 | -0.1749 | 0.0091 |  |  | 0.018 | 0.1197 | 2.44E-04 | 169.400 |
| 7 | rs11550699 | 2 | G | A | 0.3469 | -0.0195 | 0.0027 |  |  | -0.0229 | 0.0387 | 1.12E-03 | 52.160 |
| 8 | rs115700531 | 2 | C | T | 0.0209 | -0.1237 | 0.0101 |  |  | 0.217 | 0.1517 | 2.43E-04 | 150.002 |
| 9 | rs116241540 | 2 | A | G | 0.0268 | -0.0986 | 0.0097 |  |  | -0.0627 | 0.0954 | 2.66E-04 | 103.326 |
| 10 | rs11636917 | 15 | C | T | 0.3926 | 0.0173 | 0.0027 |  |  | 0.0614 | 0.039 | 1.21E-03 | 41.055 |
| 11 | rs1260326 | 2 | C | T | 0.4105 | 0.0184 | 0.0027 |  |  | -0.0487 | 0.0399 | 1.21E-03 | 46.442 |
| 12 | rs1661052 | 11 | A | G | 0.0885 | -0.0522 | 0.0044 |  |  | -0.0496 | 0.0711 | 6.44E-04 | 140.746 |
| 13 | rs16926246 | 10 | T | C | 0.1412 | 0.0651 | 0.0038 |  |  | -0.0126 | 0.0751 | 9.43E-04 | 193.491 |
| 14 | rs1799945 | 6 | G | C | 0.172 | 0.0387 | 0.0036 |  |  | -0.042 | 0.0612 | 9.44E-04 | 115.563 |
| 15 | rs1800684 | 6 | T | A | 0.1064 | -0.0257 | 0.0037 |  |  | 0.2602 | 0.0577 | 8.08E-04 | 48.246 |
| 16 | rs2292701 | 12 | A | G | 0.0179 | 0.082 | 0.0091 |  |  | 0.2669 | 0.2228 | 4.07E-04 | 81.198 |
| 17 | rs293429 | 4 | C | T | 0.2803 | -0.0156 | 0.0029 |  |  | -0.017 | 0.0394 | 1.05E-03 | 28.937 |
| 18 | rs339969 | 15 | A | C | 0.3917 | -0.0206 | 0.0026 |  |  | 0.0473 | 0.0424 | 1.16E-03 | 62.775 |
| 19 | rs34265667 | 8 | A | G | 0.0219 | 0.0646 | 0.0071 |  |  | -0.0955 | 0.1525 | 5.04E-04 | 82.784 |
| 20 | rs36075906 | 2 | T | C | 0.0378 | -0.1113 | 0.0061 |  |  | 0.0027 | 0.0727 | 4.13E-04 | 232.913 |
| 21 | rs41318029 | 10 | A | G | 0.0149 | 0.0687 | 0.0099 |  |  | 0.1331 | 0.2742 | 3.65E-04 | 48.155 |
| 22 | rs41360247 | 2 | C | T | 0.0755 | 0.0301 | 0.0053 |  |  | -0.0107 | 0.0687 | 6.30E-04 | 32.254 |
| 23 | rs4762786 | 12 | A | C | 0.3777 | -0.0613 | 0.0027 |  |  | 0.061 | 0.0392 | 1.03E-03 | 115.458 |
| 24 | rs4762975 | 12 | G | A | 0.4583 | -0.0219 | 0.0026 |  |  | -0.063 | 0.0392 | 1.16E-03 | 70.948 |
| 25 | rs499974 | 11 | A | C | 0.171 | 0.0271 | 0.0035 |  |  | 0.0028 | 0.0456 | 9.49E-04 | 59.952 |
| 26 | rs58148580 | 4 | T | C | 0.1163 | -0.0253 | 0.0042 |  |  | 0.0466 | 0.0605 | 7.12E-04 | 36.286 |
| 27 | rs58542926 | 19 | T | C | 0.0676 | 0.0667 | 0.0048 |  |  | 0.0248 | 0.0778 | 7.49E-04 | 193.094 |
| 28 | rs61733199 | 12 | C | T | 0.0239 | 0.0674 | 0.0095 |  |  | -0.0147 | 0.0913 | 3.79E-04 | 50.335 |
| 29 | rs62084251 | 17 | A | G | 0.2147 | -0.0208 | 0.0033 |  |  | 0.0609 | 0.0446 | 9.14E-04 | 39.728 |
| 30 | rs62551047 | 9 | A | G | 0.0517 | 0.0436 | 0.0057 |  |  | 0.0674 | 0.1037 | 6.02E-04 | 58.509 |
| 31 | rs645040 | 3 | T | G | 0.2286 | -0.0485 | 0.0031 |  |  | 0.0498 | 0.0538 | 9.21E-04 | 244.771 |
| 32 | rs6719561 | 2 | T | C | 0.3439 | -0.2395 | 0.0027 |  |  | -0.0576 | 0.04 | 7.22E-04 | 186.347 |
| 33 | rs6857 | 19 | T | C | 0.165 | -0.0273 | 0.0036 |  |  | -0.0144 | 0.0482 | 8.27E-04 | 57.507 |
| 34 | rs689395 | 2 | T | C | 0.1581 | 0.0226 | 0.0034 |  |  | -0.0877 | 0.068 | 9.68E-04 | 44.183 |
| 35 | rs6963451 | 7 | G | T | 0.1233 | 0.0223 | 0.0039 |  |  | 0.0666 | 0.056 | 8.43E-04 | 32.695 |
| 36 | rs72850012 | 11 | T | C | 0.0189 | 0.0504 | 0.0088 |  |  | -0.24 | 0.2057 | 3.96E-04 | 32.802 |
| 37 | rs738409 | 22 | G | C | 0.2256 | 0.018 | 0.0031 |  |  | -0.002 | 0.0453 | 1.05E-03 | 33.715 |
| 38 | rs74608404 | 2 | G | A | 0.0338 | 0.2539 | 0.007 |  |  | -0.2303 | 0.1969 | 7.47E-04 | 131.617 |
| 39 | rs75528846 | 2 | A | G | 0.0308 | -0.1155 | 0.0075 |  |  | 0.022 | 0.1226 | 3.33E-04 | 237.160 |
| 40 | rs773512 | 9 | A | G | 0.2594 | -0.0258 | 0.0029 |  |  | 0.0375 | 0.041 | 1.03E-03 | 79.149 |
| 41 | rs79208193 | 2 | T | G | 0.0547 | -0.1558 | 0.0062 |  |  | 0.2019 | 0.1006 | 3.72E-04 | 231.468 |
| 42 | rs79260437 | 2 | T | C | 0.0149 | -0.0853 | 0.0134 |  |  | -0.1981 | 0.1169 | 1.98E-04 | 40.522 |
| 43 | rs80215559 | 6 | C | T | 0.0427 | 0.0601 | 0.0049 |  |  | -0.1171 | 0.0988 | 7.24E-04 | 150.438 |
| 44 | rs8038465 | 15 | T | C | 0.4215 | 0.0199 | 0.0026 |  |  | 0.0254 | 0.0401 | 1.26E-03 | 58.581 |
| 45 | rs857721 | 1 | A | T | 0.2763 | -0.0259 | 0.0029 |  |  | -0.0249 | 0.0403 | 1.03E-03 | 79.763 |
| 46 | rs879665 | 2 | A | G | 0.1352 | 0.1191 | 0.0038 |  |  | 0.0509 | 0.0636 | 1.05E-03 | 282.328 |
| 47 | rs9393921 | 6 | G | T | 0.4463 | 0.0191 | 0.0026 |  |  | -0.2115 | 0.0399 | 1.26E-03 | 53.966 |
| 1 | rs10463891 | 5 | G | A | 0.3946 |  |  | 0.018 | 0.0025 | -0.0388 | 0.0382 | 1.30E-03 | 51.840 |
| 2 | rs1047891 | 2 | A | C | 0.3012 |  |  | 0.0249 | 0.0026 | 0.0144 | 0.0408 | 1.27E-03 | 91.717 |
| 3 | rs111226561 | 2 | T | C | 0.0129 |  |  | -0.0824 | 0.0116 | 0.0035 | 0.1176 | 2.30E-04 | 50.459 |
| 4 | rs11187157 | 10 | C | T | 0.4274 |  |  | 0.015 | 0.0024 | 0.0138 | 0.0389 | 1.35E-03 | 39.063 |
| 5 | rs1135688 | 17 | C | T | 0.336 |  |  | 0.0154 | 0.0026 | 0.1054 | 0.0451 | 1.25E-03 | 35.083 |
| 6 | rs114252547 | 2 | G | T | 0.0328 |  |  | 0.302 | 0.0065 | 0.0416 | 0.1217 | 8.85E-04 | 215.675 |
| 7 | rs114667609 | 2 | G | T | 0.0189 |  |  | -0.1956 | 0.0083 | 0.018 | 0.1197 | 2.56E-04 | 255.369 |
| 8 | rs115700531 | 2 | C | T | 0.0209 |  |  | -0.1427 | 0.0092 | 0.217 | 0.1517 | 2.57E-04 | 240.587 |
| 9 | rs11601507 | 11 | A | C | 0.0676 |  |  | 0.0293 | 0.0046 | -0.0027 | 0.0718 | 7.25E-04 | 40.571 |
| 10 | rs116241540 | 2 | A | G | 0.0268 |  |  | -0.1099 | 0.0089 | -0.0627 | 0.0954 | 2.84E-04 | 152.481 |
| 11 | rs11636917 | 15 | C | T | 0.3926 |  |  | 0.0276 | 0.0025 | 0.0614 | 0.039 | 1.33E-03 | 121.882 |
| 12 | rs11683286 | 2 | C | T | 0.2724 |  |  | -0.0197 | 0.0027 | 0.0576 | 0.044 | 1.12E-03 | 53.236 |
| 13 | rs117428098 | 12 | G | A | 0.0308 |  |  | -0.0586 | 0.0075 | -0.1542 | 0.1302 | 3.73E-04 | 61.048 |
| 14 | rs117552525 | 12 | A | G | 0.0318 |  |  | 0.0587 | 0.0069 | -0.1628 | 0.1377 | 5.13E-04 | 72.373 |
| 15 | rs117907734 | 12 | C | T | 0.0477 |  |  | 0.0276 | 0.005 | -0.1251 | 0.1072 | 6.65E-04 | 30.470 |
| 16 | rs12239046 | 1 | C | T | 0.3539 |  |  | -0.0176 | 0.0025 | 0.1113 | 0.0393 | 1.21E-03 | 49.562 |
| 17 | rs1231204 | 8 | C | G | 0.0527 |  |  | -0.0308 | 0.0056 | 0.0214 | 0.0856 | 5.28E-04 | 30.250 |
| 18 | rs12515233 | 5 | C | A | 0.161 |  |  | 0.0178 | 0.0031 | 0.0191 | 0.0526 | 1.05E-03 | 32.970 |
| 19 | rs12670798 | 7 | C | T | 0.2187 |  |  | 0.0231 | 0.0028 | 0.0115 | 0.0441 | 1.18E-03 | 68.063 |
| 20 | rs13302475 | 9 | A | G | 0.2018 |  |  | -0.0196 | 0.0031 | -0.0497 | 0.0485 | 9.76E-04 | 39.975 |
| 21 | rs13423701 | 2 | C | T | 0.1928 |  |  | -0.0226 | 0.0029 | 0.0677 | 0.0407 | 1.04E-03 | 60.732 |
| 22 | rs1490384 | 6 | T | C | 0.493 |  |  | -0.0189 | 0.0024 | -0.0284 | 0.038 | 1.26E-03 | 62.016 |
| 23 | rs1661052 | 11 | A | G | 0.0885 |  |  | -0.0556 | 0.0041 | -0.0496 | 0.0711 | 6.87E-04 | 183.900 |
| 24 | rs16926246 | 10 | T | C | 0.1412 |  |  | 0.0768 | 0.0035 | -0.0126 | 0.0751 | 1.05E-03 | 211.489 |
| 25 | rs17863840 | 2 | T | C | 0.0119 |  |  | -0.1436 | 0.0129 | -0.0449 | 0.1055 | 1.83E-04 | 123.917 |
| 26 | rs17868337 | 2 | A | G | 0.0308 |  |  | -0.2259 | 0.0079 | -0.091 | 0.1306 | 2.54E-04 | 217.670 |
| 27 | rs1799945 | 6 | G | C | 0.172 |  |  | 0.0477 | 0.0034 | -0.042 | 0.0612 | 1.02E-03 | 196.824 |
| 28 | rs2193045 | 12 | G | A | 0.2704 |  |  | -0.0147 | 0.0026 | 0.0489 | 0.0391 | 1.17E-03 | 31.966 |
| 29 | rs2292701 | 12 | A | G | 0.0179 |  |  | 0.0939 | 0.0085 | 0.2669 | 0.2228 | 4.47E-04 | 122.038 |
| 30 | rs2391175 | 7 | G | A | 0.3688 |  |  | 0.0151 | 0.0026 | 0.0226 | 0.0396 | 1.25E-03 | 33.729 |
| 31 | rs2657879 | 12 | G | A | 0.2127 |  |  | 0.0173 | 0.0031 | 0.0546 | 0.0493 | 1.05E-03 | 31.144 |
| 32 | rs2748427 | 17 | G | A | 0.2127 |  |  | -0.0198 | 0.0029 | -0.1163 | 0.0475 | 1.04E-03 | 46.616 |
| 33 | rs2792751 | 10 | C | T | 0.3082 |  |  | -0.0216 | 0.0027 | -0.0657 | 0.0407 | 1.12E-03 | 64.000 |
| 34 | rs2909210 | 17 | T | G | 0.4423 |  |  | 0.0148 | 0.0024 | 0.0418 | 0.0392 | 1.35E-03 | 38.028 |
| 35 | rs2974338 | 8 | G | A | 0.3877 |  |  | 0.0182 | 0.0025 | 0.0218 | 0.0395 | 1.30E-03 | 52.998 |
| 36 | rs3131283 | 6 | C | T | 0.1054 |  |  | -0.0312 | 0.0034 | 0.2602 | 0.0577 | 8.69E-04 | 84.208 |
| 37 | rs340005 | 15 | A | G | 0.3867 |  |  | -0.0262 | 0.0025 | 0.0529 | 0.0425 | 1.19E-03 | 109.830 |
| 38 | rs34664882 | 8 | A | G | 0.0219 |  |  | 0.0726 | 0.0066 | -0.0932 | 0.1527 | 5.51E-04 | 121.000 |
| 39 | rs3794271 | 12 | A | G | 0.3648 |  |  | 0.025 | 0.0024 | -0.0975 | 0.0391 | 1.38E-03 | 108.507 |
| 40 | rs4149056 | 12 | C | T | 0.161 |  |  | 0.1882 | 0.0033 | 0.0134 | 0.0471 | 1.39E-03 | 252.455 |
| 41 | rs4415806 | 11 | C | T | 0.4264 |  |  | 0.0135 | 0.0024 | -0.004 | 0.0402 | 1.35E-03 | 31.641 |
| 42 | rs4643532 | 2 | C | T | 0.162 |  |  | -0.0382 | 0.0034 | 0.0284 | 0.0514 | 8.57E-04 | 126.232 |
| 43 | rs4787458 | 16 | G | A | 0.3231 |  |  | -0.0186 | 0.0024 | 0.024 | 0.0381 | 1.26E-03 | 60.063 |
| 44 | rs4820268 | 22 | A | G | 0.4225 |  |  | 0.0274 | 0.0024 | 0.02 | 0.0384 | 1.38E-03 | 130.340 |
| 45 | rs499974 | 11 | A | C | 0.171 |  |  | 0.0286 | 0.0033 | 0.0028 | 0.0456 | 1.01E-03 | 75.111 |
| 46 | rs56001387 | 2 | G | A | 0.2177 |  |  | -0.0159 | 0.0029 | 0.0122 | 0.0453 | 1.05E-03 | 30.061 |
| 47 | rs58148580 | 4 | T | C | 0.1163 |  |  | -0.0253 | 0.0039 | 0.0466 | 0.0605 | 7.67E-04 | 42.083 |
| 48 | rs58542926 | 19 | T | C | 0.0676 |  |  | 0.0402 | 0.0045 | 0.0248 | 0.0778 | 7.58E-04 | 79.804 |
| 49 | rs61733199 | 12 | C | T | 0.0239 |  |  | 0.0674 | 0.0088 | -0.0147 | 0.0913 | 4.09E-04 | 58.662 |
| 50 | rs62020698 | 15 | T | C | 0.1004 |  |  | -0.0243 | 0.0042 | -0.1498 | 0.1204 | 7.14E-04 | 33.474 |
| 51 | rs62084251 | 17 | A | G | 0.2147 |  |  | -0.0198 | 0.003 | 0.0609 | 0.0446 | 1.01E-03 | 43.560 |
| 52 | rs62191634 | 2 | T | C | 0.0258 |  |  | 0.0416 | 0.0064 | 0.0495 | 0.0958 | 5.34E-04 | 42.250 |
| 53 | rs62551047 | 9 | A | G | 0.0517 |  |  | 0.045 | 0.0053 | 0.0674 | 0.1037 | 6.50E-04 | 72.090 |
| 54 | rs62576295 | 9 | A | G | 0.3012 |  |  | -0.0179 | 0.0026 | -0.001 | 0.0424 | 1.17E-03 | 47.398 |
| 55 | rs645040 | 3 | T | G | 0.2286 |  |  | -0.0542 | 0.0028 | 0.0498 | 0.0538 | 1.01E-03 | 274.699 |
| 56 | rs6682423 | 1 | T | C | 0.3111 |  |  | 0.0212 | 0.0025 | 0.0225 | 0.0407 | 1.31E-03 | 71.910 |
| 57 | rs6729339 | 2 | A | G | 0.2793 |  |  | 0.0149 | 0.0026 | -0.0601 | 0.0464 | 1.25E-03 | 32.842 |
| 58 | rs6756943 | 2 | A | G | 0.2922 |  |  | 0.0193 | 0.0026 | 0.0427 | 0.0447 | 1.26E-03 | 55.102 |
| 59 | rs676388 | 19 | C | T | 0.4702 |  |  | 0.0209 | 0.0024 | 0.0492 | 0.0387 | 1.37E-03 | 75.835 |
| 60 | rs689395 | 2 | T | C | 0.1581 |  |  | 0.0219 | 0.0032 | -0.0877 | 0.068 | 1.03E-03 | 46.837 |
| 61 | rs6963451 | 7 | G | T | 0.1233 |  |  | 0.0219 | 0.0036 | 0.0666 | 0.056 | 9.13E-04 | 37.007 |
| 62 | rs7249084 | 19 | C | T | 0.4732 |  |  | 0.0132 | 0.0024 | 0.0242 | 0.0381 | 1.35E-03 | 30.250 |
| 63 | rs728160 | 4 | C | T | 0.4026 |  |  | 0.0135 | 0.0024 | 0.0106 | 0.0397 | 1.35E-03 | 31.641 |
| 64 | rs72850012 | 11 | T | C | 0.0189 |  |  | 0.046 | 0.0082 | -0.24 | 0.2057 | 4.21E-04 | 31.469 |
| 65 | rs738409 | 22 | G | C | 0.2256 |  |  | 0.0184 | 0.0029 | -0.002 | 0.0453 | 1.13E-03 | 40.257 |
| 66 | rs74608404 | 2 | G | A | 0.0338 |  |  | 0.3088 | 0.0066 | -0.2303 | 0.1969 | 8.84E-04 | 189.106 |
| 67 | rs75560791 | 2 | A | G | 0.0477 |  |  | -0.1467 | 0.0048 | 0.0128 | 0.0705 | 4.89E-04 | 234.066 |
| 68 | rs773512 | 9 | A | G | 0.2594 |  |  | -0.03 | 0.0027 | 0.0375 | 0.041 | 1.10E-03 | 123.457 |
| 69 | rs78936963 | 2 | G | A | 0.0229 |  |  | 0.3599 | 0.0074 | -0.151 | 0.0767 | 8.73E-04 | 236.376 |
| 70 | rs7907754 | 10 | G | A | 0.0915 |  |  | -0.0425 | 0.004 | 0.0472 | 0.0526 | 7.22E-04 | 112.891 |
| 71 | rs79208193 | 2 | T | G | 0.0547 |  |  | -0.1856 | 0.0056 | 0.2019 | 0.1006 | 3.88E-04 | 108.449 |
| 72 | rs80215559 | 6 | C | T | 0.0427 |  |  | 0.0659 | 0.0046 | -0.1171 | 0.0988 | 7.80E-04 | 205.237 |
| 73 | rs8038465 | 15 | T | C | 0.4215 |  |  | 0.0154 | 0.0024 | 0.0254 | 0.0401 | 1.35E-03 | 41.174 |
| 74 | rs8187707 | 10 | T | C | 0.0696 |  |  | -0.0313 | 0.0052 | -0.1805 | 0.122 | 5.68E-04 | 36.231 |
| 75 | rs8330 | 2 | C | G | 0.2366 |  |  | 0.1733 | 0.0028 | 0.0206 | 0.056 | 1.59E-03 | 183.726 |
| 76 | rs857721 | 1 | A | T | 0.2763 |  |  | -0.0343 | 0.0027 | -0.0249 | 0.0403 | 1.09E-03 | 161.384 |
| 77 | rs879665 | 2 | A | G | 0.1352 |  |  | 0.1436 | 0.0035 | 0.0509 | 0.0636 | 1.20E-03 | 168.344 |
| 78 | rs900400 | 3 | C | T | 0.3897 |  |  | -0.0147 | 0.0025 | 0.0139 | 0.0409 | 1.22E-03 | 34.574 |
| 79 | rs9438866 | 1 | C | A | 0.1561 |  |  | 0.02 | 0.0034 | -0.0524 | 0.0475 | 9.63E-04 | 34.602 |
| 80 | rs9914988 | 17 | A | G | 0.2018 |  |  | -0.0187 | 0.0029 | 0.0134 | 0.0482 | 1.04E-03 | 41.580 |

Note: These SNPs are associated with serum bilirubin at the genome-wide significance level (p < 5.0×10^-8^).

SNP: single-nucleotide polymorphism; Chr, chromosome; EA, effect allele; OA, other allele; EAF, effect allele frequency; Beta, SNP effect size; SE, standard error; R^2^, percentage of the variation explained per allele; F, F-statistic.
