# Supplementary material for: Causal inference between serum bilirubin levels and juvenile idiopathic arthritis‐associated uveitis: A bidirectional Mendelian randomization study
Source: Health Sci Rep. 2024 Feb 1;7(2):e1847. doi: 10.1002/hsr2.1847 (PMC10835017; doi:10.1002/hsr2.1847)
Supplement: Supplementary file 2 — Supporting information. [file HSR2-7-e1847-s001.docx]

Supplement Table 2. Summary statistics for MR analysis of the potential causal effect of JIA associated iridocyclitis on serum bilirubin levels.

| No. | SNP | Chr. | EA | OA | EAF | Exposure: iridocyclitis | | Outcome: direct bilirubin | | Outcome: total bilirubin | | R^2^ | F |
| --- | --- | --- | --- | --- | --- | --- | --- | --- | --- | --- | --- | --- | --- |
|  |  |  |  |  |  | Beta | SE | Beta | SE | Beta | SE |  |  |
| 1 | rs115628514 | 6 | C | A | 0.0467 | -1.6108 | 0.0954 | -0.0128 | 0.0076 |  |  | 4.44E-06 | 285.093 |
| 2 | rs115999992 | 6 | A | T | 0.0467 | 2.2251 | 0.0863 | -0.0118 | 0.0068 |  |  | 1.04E-02 | 266.779 |
| 3 | rs116324499 | 6 | C | T | 0.0159 | 0.305 | 0.044 | 0.0177 | 0.0099 |  |  | 4.44E-04 | 48.050 |
| 4 | rs2252711 | 6 | C | T | 0.2425 | 0.4104 | 0.043 | 0.0052 | 0.0031 |  |  | 5.61E-04 | 91.091 |
| 5 | rs2524229 | 6 | G | T | 0.0149 | 0.295 | 0.0521 | -0.0248 | 0.0089 |  |  | 3.67E-04 | 32.060 |
| 6 | rs62395309 | 6 | G | A | 0.0119 | 0.2758 | 0.0442 | -0.0028 | 0.0118 |  |  | 4.17E-04 | 38.935 |
| 7 | rs76389676 | 6 | G | A | 0.0398 | -0.5789 | 0.0987 | 0.0126 | 0.0058 |  |  | 3.38E-05 | 34.401 |
| 1 | rs115628514 | 6 | C | A | 0.0467 | -1.6108 | 0.0954 |  |  | -0.0126 | 0.0071 | 4.44E-06 | 285.093 |
| 2 | rs115999992 | 6 | A | T | 0.0467 | 2.2251 | 0.0863 |  |  | -0.0192 | 0.0063 | 1.04E-02 | 264.779 |
| 3 | rs116324499 | 6 | C | T | 0.0159 | 0.305 | 0.044 |  |  | 0.0196 | 0.0092 | 4.44E-04 | 48.050 |
| 4 | rs2252711 | 6 | C | T | 0.2425 | 0.4104 | 0.043 |  |  | 0.0031 | 0.0029 | 5.61E-04 | 91.091 |
| 5 | rs2524229 | 6 | G | T | 0.0149 | 0.295 | 0.0521 |  |  | -0.0115 | 0.0082 | 3.67E-04 | 32.060 |
| 6 | rs62395309 | 6 | G | A | 0.0119 | 0.2758 | 0.0442 |  |  | 0.0003 | 0.0109 | 4.17E-04 | 38.935 |
| 7 | rs76389676 | 6 | G | A | 0.0398 | -0.5789 | 0.0987 |  |  | 0.0116 | 0.0054 | 3.38E-05 | 34.401 |

Note: These SNPs are associated with iridocyclitis at the genome-wide significance level (p < 5.0×10^-8^).

SNP: single-nucleotide polymorphism; Chr, chromosome; EA, effect allele; OA, other allele; EAF, effect allele frequency; Beta, SNP effect size; SE, standard error; R^2^, percentage of the variation explained per allele; F, F-statistic.
